# Deep Brain Stimulation in Persistent Vegetative States: Ethical Issues Governing Decision Making

**DOI:** 10.1155/2014/641213

**Published:** 2014-03-16

**Authors:** Sara Patuzzo, Paolo Manganotti

**Affiliations:** ^1^Department of Public Health and Community Medicine, Unit of Forensic Medicine, University of Verona, Piazzale L. A. Scuro 10, 37134 Verona, Italy; ^2^Department of Neurologic and Movement Sciences, Unit of Neurology, University of Verona, Piazzale L. A. Scuro 10, 37134 Verona, Italy

## Abstract

The aim of the present paper was to investigate the fundamental ethical issues of Deep Brain Stimulation (DBS) on patients remaining in Persistent Vegetative State (PVS). First, the purpose of this analysis was to discuss the nature of this intervention in order to classify it such as an ordinary clinical practice, or otherwise as an extraordinary clinical practice or as experimental research. Second, ethical issues, criticisms, and methodological issues of this intervention, also in the future perspectives, are discussed, attempting to identify who could give informed consent for a patient in PVS.

## 1. Background

A Persistent Vegetative State (PVS) refers to a disorder of consciousness in which severely brain-injured patients remain in a state of wakefulness without detectable awareness. In this extended state of unconsciousness, accompanied by nearly normal cycles of sleeping and waking, the brainstem and thalamus are relatively spared, but cortical functional connectivity is limited or absent. Persistently vegetative individuals have no signs of awareness of themselves or their environment. Some may progress to a permanent Vegetative State (VS), generally 3 months after an anoxic brain event and 12 months after brain trauma, while others may progress to a Minimally Conscious State (MCS), in which integrated but under sustained cortical functions are retained [1, 2]. If the disorder persists for longer than 12 months after severe traumatic brain injury, the state is generally considered to be immutable and no treatment has been shown to accelerate recovery or improve functional outcome [3, 4]. Nonetheless, some studies have shown unexpected preservation of large-scale cerebral networks in MCS patients, a condition characterized by definite behavioral evidence of awareness of self or the environment [5–9].

Neurostimulation to restore cognitive and physical functions is an innovative and promising technique for treating patients with severe brain injury. Deep Brain Stimulation (DBS) has been proposed as an experimental therapeutic strategy that might produce consistent and sustained effects of maintaining excitatory activity within functionally disconnected forebrain neurons (2012). It is used in treating in several neurological and psychiatric diseases. Besides its invasiveness associated with surgical risks and complications, another major barrier to its wider use, is the syndromic heterogeneity and variance of subjects who might benefit from DBS. Furthermore, the selection of potential recipients of DBS is limited by the current inability to estimate cerebral function based on bedside examination [4].

## 2. Deep Brain Stimulation: Clinical Practice and Experimental Research

Deep Brain Stimulation (DBS) is a nonpharmacologic method to stimulate electrically the deep structures of human brain. DBS in clinical practice is used as a therapy to treat neurological disorders like Parkinson, essential tremor, or dystonia. DBS in scientific research is explored to treat psychiatric disorders like major depression, obsessive-compulsive disorders, addiction, or obesity. Despite the large clinical applications, the precise mechanism of action of DBS remains uncertain and different and multiple hypothetical mechanisms are reported [10].

Particular fields of clinical applications of DBS are the studies conducted on severely brain-injured patients, like patients remaining in the chronic Minimally Conscious State (MCS) or in the Persistent Vegetative State (PVS) [11]. MCS is a clinical condition which differs from PVS by the presence of inconsistent, but clearly discernible, behavioural evidence of consciousness [12]: it can be defined as ≪a condition of severely altered consciousness in which minimal but definite behavioural evidence of self or environmental awareness is demonstrated≫ [13]. PVS can be defined as a wakeful unconscious state or Vegetative State (VS) that lasts longer than four weeks, whereas the diagnosis of permanence is classified after approximately one year.

The introduction and the possible therapeutic applications of DBS in these disorders of consciousness have discovered an amount of ethical issues on different perspectives. About its real clinical utility defined on the evidence based medicine, it is also necessary to discuss who could give the informed consent to DBS, particularly if the patient is incompetent.

## 3. Neuroethics and Ethical Issues of DBS

Bioethicists and philosophers have been discussing DBS both in care and in research projects to identify related fundamental ethical issues [14, 15]. The common first major topic could be the controversial possibility, with neural interventions, to alter the experience of personhood [16, 17].

### 3.1. Ethical Issues of DBS in Clinical Research

The most important ethical challenges of DBS in clinical treatment are described as follows:

- balancing associated risks, side effects, complications, and benefits;
- the fair and careful selection of patients: candidates should be “resistant” patients from other treatments. Special attention should be given in order to protect and safeguard the health, the rights, and the interests of vulnerable groups of patients, like children or adolescents in pediatric treatment with DBS;
- the clear and adequate communication to patients of the information regarding possible risks and realistic expectations;
- the respect of patients' autonomy expressed by the informed consent or dissent to DBS, such as for any medical intervention;
- the principle of Justice with regard to resources allocation for DBS treatments.

### 3.2. Ethical Issues of DBS in the Perspective of Scientific Research

The several ethical implications of DBS in experimental interventions are listed as follows:

- the meaning and the value of the research itself;
- the proportional and acceptable ratio of risks for research subjects and potential benefits also in the interest of future patients;
- which participants should be included or excluded in the experiments: research with DBS should be used when other procedure options have been exhausted;
- promoting high-quality methods of scientific research;
- the participants' valid informed consent to the research project, the prompt communication concerning eventual changes of the experiment, and the respect of their possibility to dissent at any time;
- monitoring the safety, the health, and the wellbeing of participants during the research time;
- the respect of their privacy;
- the ethical discussion regarding DBS applications with cognitive human enhancement purpose [18];
- the transparency and the independence of the assessment in order to avoid conflict of interests.

## 4. DBS on the PVS: Ethical Discussion

The most important ethical issue of DBS on PVS is the patient's* incompetence* to consent or dissent to treatment. Preliminarily to the examination of the core of the matter, the meanings of the concept of “informed consent” and of “medical paternalism” are presented below.

### 4.1. The Medical Paternalism Model

Especially in Latin Mediterranean countries of Europe, until the second part of the 20th century, the medical practice has been grounded on a profound paternalism. The physician decided the medical interventions for his patients, such as that a father decides what is right or wrong for the good of his sons.

The fundamental moral principles of medical paternalism are the* principle of beneficence* (which guides the ethical theory for physicians to do good for the patient in his best interests) and the* principle of nonmaleficence* (which states that the physician must refrain from doing harm toward patient).

### 4.2. The Informed Consent Model

Especially after the Second World War and the knowledge of the Nazi human experiments, biomedical ethics in laws [19] and in clinical practice moved from medical paternalism ethics to informed consent model that expresses the right of the patient to decide himself to consent or dissent to diagnostic or therapeutic interventions proposed by the physician. In the health care setting, the informed consent process is structured into two phases.

- In the first moment the physician provides the patient with the information, in a way that the patient could understand, regarding the diagnosis, the nature and the purpose of proposed procedure, the related risks and benefits, the alternative options, and the consequences of not undergoing the proposed treatment and its alternative possibilities.
- In the second moment the patient expresses his voluntary authorization or agreement to proceed and to undergo the specific medical intervention, or his decision to refuse it.

The moral foundation of the informed consent procedure is the bioethical* principle of autonomy*, which suggests the physician's duty to respect the patient's right to self-determination.

If the person is* competent*, he owns the ability to give his informed consent or dissent to clinical treatments as patient, or to research experiments as participant. In fact, to give a valid informed consent or dissent to medical intervention both in clinical practice and in research project, a requirement of competency must be present.

There are two different rules of procedure to collect the consent to medical interventions in case of ordinary clinical practice and in the other cases of extraordinary clinical practice or of scientific research.

- In the case of* ordinary clinical practice*, which constitutes the basic care aimed at treating a clinical condition, the principles of beneficence and nonmaleficence guide the physician in his clinical decisions. In fact, so long a medical treatment does not have a significant potential for harmful outcomes, patient's informed consent is not perceived to be necessary.
- In the other case of* extraordinary clinical practice*, which outlines a type of intervention that is usually highly invasive and might be considered burdensome to the patient, the incompetent patient should be protected in his best interests by an authorized legal representative (appointed by the judge supervising cases concerning guardianship), with the authority to give the informed consent or dissent on behalf of him, in cooperation with the treating doctor. In Italy this is also stated by the Italian Civil Code (Italian Civil Code, art. 414.) and by the Code of good medical practice or conduct, named “Code of medical deontology” (Italian Code of medical deontology 2006, art. 37.) In this frame, taking also into consideration that many European countries developed laws concerning the “advance health care directive or declaration” (e.g., France, Germany, Netherlands, Switzerland, and England.), that is, the so-called “Living will”: a document that the person uses to set out the medical care that he wants or does not want in the event that he becomes incapable of communicating his wishes. The common legal basis is the European Oviedo Convention on Human Rights and Biomedicine (see [18]).

Article 9, “Previously expressed wishes”: ≪The previously expressed wishes relating to a medical intervention by a patient who is not, at the time of the intervention, in a state to express his or her wishes shall be taken into account≫.

In Italy a law on Living will (already mentioned by the Code of medical deontology (Italian Code of medical deontology 2006, artt. 35, 38.)) has been under discussion [20].

In the case of medical interventions in* scientific research* (intended as administration of medical products for clinical trial), in addition, a previous screening by a local ethics committee is necessary [21].

### 4.3. DBS on PVS: Who Gives Informed Consent?

As mentioned above, presumably the most troubling aspect in medical ethics of DBS on PVS is the impossibility for the patient, who is incapable of giving his consent to the practice.

But DBS on PVS should be intended as an* ordinary clinical practice*, or otherwise as an* extraordinary clinical practice* or as a* scientific research?*

The PVS is a chronic neurological disorder of consciousness characterized by wakefulness without awareness. It can be intended as a special clinical condition produced by medical interventions. Hence, such as for any other medical interventions, PVS need a “start-informed consent,” which could be obtained by

- the patient himself, previously discerning his personally expressed wishes when he was still capable, or eventually following his advance directive (“Living will”);
- each legal patient representative or surrogate.

In case of emergency or without an explicit dissent of the patient or of his legal representative, the physician could be the subject of the decision.

In other words, if a patient remain in a PVS (and measures have not been set in order to interrupt life-sustaining treatments), a previous explicit or implied consent has been given. Hence, any ordinary medical procedures and nursing care, used to monitor and keep the clinical condition of the PVS hoping to find care to treat the patient, do not need further consent. On the contrary, in the PVS, procedures of treatment interruption (the dissent to PVS itself) or of corrupting physical and mental integrity could be intended as extraordinary interventions; therefore they need a previous consent.

The DBS in PVS could be considered an* ordinary medical intervention*, because it is neither an interruption treatment nor a treatment which entails risks of corrupting (physical and) mental integrity, since in PVS the human brain is already severely damaged.

In addition, it should be taken into consideration that, on the one hand, in the special clinical condition of PVS, it is difficult to be making a worse state.

DBS could not be exclusively regarded as a research experiment, but, rather, a* clinical attempt at therapeutic treatment with research aspects*.

In conclusion, in case of DBS for PVS, and only under such circumstance, this medical intervention could be described as an ordinary care treatment, which does not call for patient's consent, and as a clinical attempt at therapeutic treatment (with research aspects), which does not call for a previous screening by a local ethics committee, but only maybe this could be* informed* of the DBS starting intervention.

When DBS is applied to a patient in PVS, the hope for a health benefit for the patient outweighs the risks connected to DBS intervention. By this way, there is the full respect of both biomedical ethical principles of beneficence and of non-maleficence. In addition, there is not conflict with the ethical principle of patient's autonomy, because it is already respected in the beginning of PVS clinical condition, and afterwards it is confirmed by its persisting state. By this perspective, the intervention of DBS on a patient in PVS could be applied respecting the ethical principles that inform it, such as that inform other fields of medical treatment and research: firstly, beneficence, nonmaleficence, and autonomy [20].

Our conclusion is that DBS for PVS does not require informed consent, because

- PVS can be intended as a special clinical condition produced by medical interventions. The pathological problem lies in the brain status;
- such as for any other medical interventions, PVS needs a “start-informed consent”;
- if a patient is in PVS, we could presume that the PVS started with a previous informed consent, or in a case of emergency by a decision of the physician;
- if a patient persisting in the PVS clinical condition, it is because his legal representative does not ask for the interruption of the medical interventions that implement this clinical condition, otherwise not even the patient himself asked for that interruption by “advanced directives” (“Living will”);
- if the patient persisting in PVS, any medical intervention necessary to the PVS itself or to treat the main problem—the brain status, for example, with a DBS intervention—does not need further consent.

## 5. Noninvasive Brain Stimulation Techniques: A Possible Future Tool for Testing the Candidate for DBS

Among currently available noninvasive painless stimulation techniques, single-pulse Transcranial Magnetic Stimulation (TMS) has been demonstrated to be effective for assessing motor cortex excitability and the integrity of conduction along the central and peripheral motor pathways. Similarly, repetitive Transcranial Magnetic Stimulation (rTMS) has been shown to induce prolonged functional changes in cerebral cortex in normal conditions and therapeutic effects in different diseases [22]. Several studies suggest that the thalamocortical system can be engaged in rapid causal interactions [23–26]. One way to study this phenomenon is to perturb directly a subset of cortical neurons with TMS and monitor the brain's reaction using electroencephalography (EEG) [27–31].

To date, few studies have focused on the use of TMS in patients with impaired consciousness [32–35]. Recent advances in EEG-TMS coregistration have shed new light on EEG reactivity in humans [36, 37]. For instance, Babiloni et al. demonstrated a relationship between alpha EEG rhythm and conscious awareness. They showed that the parietal and occipital source power of alpha rhythm was high in the normal subjects, low in the PVS patients who recovered some level of consciousness at 3-month followup, and practically null in the PVS patients who did not recover. Their findings suggest that the sources of alpha rhythm are related to the outcome of PVS patients at 3-month followup. Corroborating this hypothesis, our recent study reported the reactivity of a single MCS patient after brain stimulation, in which an increase in the alpha band was correlated with functional improvement [38]. Piccione et al. (2011) and Manganotti et al. (2013) reported results of a 20 Hz rTMS protocol applied to MCS patients. They highlighted the therapeutic effect of rTMS concluding that thirty applications of rTMS protocol may promote clinically significant neurobehavioral recovery in chronic severe traumatic brain injury [39, 40].

Generally, in behaviorally awake but unresponsive VS patients, TMS triggers a simple, local slow response that indicates a breakdown in effective connectivity, similar to that observed in unconscious sleeping or anaesthetized patients. In contrast, in MCS patients, who show fluctuating signs of nonreflexive behavior, TMS seems to trigger complex activations that sequentially involve distant cortical areas ipsilateral and contralateral to the site of stimulation.

Evidence from electrophysiological studies of stimulation over a healthy primary motor cortex (M1) suggests that there is a progressive increase in the excitability of local circuits during rTMS, but not only. Remote changes in cortical and subcortical activity, including associative regions such as the thalamus, caudate nucleus, and putamen, may be involved in stimulation. The nature of the remote effect of TMS is not well understood. The presumed net facilitatory effect on neural activity in remote regions may be produced by transsynaptic or direct activation of corticocortical or corticosubcortical neurons.

The possibility to use noninvasive brain stimulation techniques in this disease could help to validate the issue of brain stimulation but, most importantly, could be a test based on the clinical based medicine to investigate the possible candidates for implanted stimulator. TMS is only one of the methods, the most known and the most validated. Probably we need more studies and clinical trials. The noninvasivity of this technique lets possible large future studies in this direction. Other future techniques as the theta burst with TMS, the Transcranial Direct Current Stimulation (TDCS), or other sensory stimulation can be and should be tested in order to improve the clinical protocols in these patients.

tDCS is another technique, minimally invasive, that involves applying weak direct current via electrodes. tDCS differs from TMS because it only allows for neuromodulation and not neurostimulation [41] and because the current applied to the brain is insufficient to induce the rapid shift in neuronal membrane potentials required to produce action potentials [42].

## 6. Conclusions

If the ethical analysis about DBS on PVS discussed in the present paper [43] is correct, it could be cogent and maybe much more, for these types of stimulation techniques (TMS and tDCS), precisely for their non- or less invasive feature.
